# A192 EFFICACY OF USTEKINUMAB IN SMALL BOWEL STRICTURES OF FIBROSTENOTIC CROHN'S DISEASE AS ASSESSED BY INTESTINAL ULTRASOUND

**DOI:** 10.1093/jcag/gwac036.192

**Published:** 2023-03-07

**Authors:** J St-Pierre, R Rosentreter, A Kiraly, J Hart Szostakiwskyj, K Novak, R Panaccione, G Kaplan, S Devlin, C Seow, R Ingram, C Ma, S Wilson, A Medellin, C Lu

**Affiliations:** 1 Department of Medicine; 2 Department of Radiology; 3 Methods and Analytics, Clinical Research Unit, University of Calgary, Calgary, Canada

## Abstract

**Background:**

Small bowel Crohn’s disease (CD) strictures can lead to debilitating obstructive symptoms and the deterioration of quality of life. Imaging modalities such as intestinal ultrasound (IUS) are invaluable in the diagnosis of strictures. The use of IUS in CD is rapidly growing, is cost-effective, easily repeatable, and similar in accuracy to magnetic resonance enterography. Evidence for medical management of fibrostenotic CD has been limited to anti-tumor necrosis factor biologics. Studies on the efficacy of other biologic therapies for strictures such as ustekinumab, a p40/interleukin 12 and 23 inhibitor, are lacking.

**Purpose:**

The objective of this study was to evaluate the efficacy of ustekinumab in the treatment of small bowel strictures on IUS.

**Method:**

This retrospective cohort study evaluated the IUS changes of terminal ileal (TI) CD strictures at baseline and 12 months following ustekinumab initiation from 2016 to 2020 at a single tertiary care center. Strictures identified were defined as 1) increased bowel wall thickness (BWT) > 3mm, 2) narrowed luminal apposition, and 3) presence of pre-stenotic dilation (PSD) or the inability to pass the colonoscope through the narrowed area. Changes in sonographic parameters (BWT, luminal size, PSD, length, hyperemia, inflammatory fat, dysfunctional peristalsis) were recorded at baseline prior to initiation of ustekinumab and compared 12 months after treatment. Differences from baseline to 12 months were paired within-person and statistical analysis was performed using paired T-tests for continuous variables and McNemar’s test for categorical variables.

**Result(s):**

Of the 18 patients identified, 55% (*n *= 10) were male, median age was 49 years (Q1-Q3: 33-63 years) at initial scan, with median CD duration of 10 years (Q1-Q3: 8-20 years). The majority of TI strictures were surgically naïve (67%, *n *= 12). Between pre- and 12-month post ustekinumab therapy scans, there was significant improvement in BWT [8.2 mm vs 7.2 mm, *p *= 0.048], however there was no significant difference in the presence of peri-enteric inflammatory fat (*p *= 0.10), mean stricture length (17.7 vs 21.7 cm, *p *= 0.18), and mean stricture lumen diameter (3.3 mm vs 2.7 mm, *p *= 0.44) (Table 1). There was also no significant difference in the presence of stricture-associated peri-enteric fat (89% vs 67%, *p *= 0.10), stricture-associated hyperemia (83% vs 89%, *p *= 0.65) or dysfunctional peristalsis (50% vs 61%, *p *= 0.41) (Table 1).

**Image:**

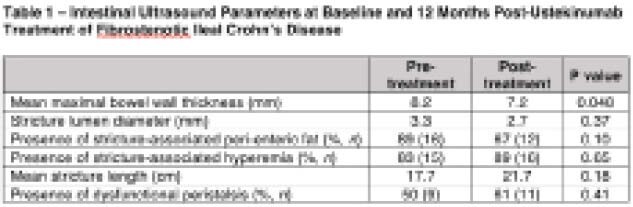

**Conclusion(s):**

Our study is the first to report the efficacy of ustekinumab in small bowel CD strictures using IUS at baseline and 12 months. This study shows that although ustekinumab leads to improvement in overall sonographic appearance of bowel thickness, it does not improve luminal narrowing nor PSD, two hallmark criteria of fibrostenosis. More extensive studies with larger sample sizes evaluating ustekinumab, or combination therapies, are required to identify their role in stricturing CD.

**Please acknowledge all funding agencies by checking the applicable boxes below:**

None

**Disclosure of Interest:**

None Declared

